# Setting the Weights: The Women’s Capabilities Index for Malawi

**DOI:** 10.1007/s11205-016-1502-3

**Published:** 2016-12-28

**Authors:** Giulia Greco

**Affiliations:** Department of Global Health and Development, London School of Hygiene and Tropical Medicine, 15-17 Tavistock Place, London, WC1H 9SH UK

**Keywords:** Capability approach, Developing country, Quality of life, Wellbeing, Multidimensional index, Weights

## Abstract

Standard indicators of wellbeing such as the QALY for health and GDP per capita for economic development have been increasingly regarded as being too narrow in focus. There is a need to develop multidimensional measures of wellbeing that encompass the full range of factors that make life worth living. This study is part of a project that aims at developing a multidimensional index based on Sen’s capability framework to assess women’s wellbeing in rural Malawi: the Women's Capabilities Index. The project identifies a set of capabilities relevant to the context; proposes a methodology to measure robustly these capabilities; aggregates the capabilities into a single metric (index); and validates and tests the index. This paper focuses on the weighting and aggregation of the index. Four weighting methods of aggregation are chosen: two normative approaches; a data-driven approach; and a hybrid method. The different methods have implications on the results which are critically assessed and compared. This study contributes to the literature on the implications of adopting different methods for setting the weights in composite measures of wellbeing.

## Introduction

There is an established consensus rooted in Rawls’ and Sen’s theories that wellbeing is intrinsically multidimensional (Rawls 1971; Sen and Nussbaum 1993; Anand and Sen 1997; McGillivray 2012).

Standard indicators of wellbeing such as the QALY for health and GDP per capita for economic development have been increasingly regarded by academics and policy makers as being too narrow in focus. They fail to address the complexity of human nature, social progress, and issues of equity (Coast 2004; Anand and Dolan 2005; Greco et al. 2016; Alkire and Santos 2013).

The ‘measuring wellbeing’ agenda calls for improved and new statistical measures, aimed at filling the gap between standard economic statistics (which are mainly focused on measuring the material wealth of people) and indicators that have a more direct bearing on people’s lives (Stiglitz et al. 2009; OECD 2011; O’Donnell et al. 2014; Diener and Suh 1997).

In order to better assess and monitor progress in society, there is a need to develop multidimensional measures of wellbeing that encompass the full range of factors that make life worth living (Stiglitz et al. 2009). Some of the ongoing work on the development of multidimensional wellbeing indicators is inspired by the capabilities approach developed by Amartya Sen (1982, 1985, 1993).

A central normative argument of Sen’s seminal work states that individual advantage should not be seen merely as opulence or utility, and should not be assessed using people’s preferences or desires, but instead in terms of the freedoms that people have to choose the kind of life they have reason to value (Sen 1985, 1993). With this in mind, Sen argues that social and public policy should aim to expand people’s capabilities, and a policy would be considered successful if it led to an expansion of people’s capability set.

Composite indicators are recognised as a useful tool in policy analysis. They can measure complex multidimensional concepts which cannot be captured by a single indicator and are easier to interpret than a series of separate indicators (Nardo et al. 2005). There are a number of challenges that need to be considered when constructing a composite measure of wellbeing: the selection of dimensions and indicators; the selection of relative weights for aggregating the dimensions; the validation of the measure. The search for appropriate weights is often perceived as the most significant challenge (Stiglitz et al. 2009; Decancq and Lugo 2012): to assess the importance of each dimension and whether and how to aggregate them.

There is a range of methods available for aggregating dimensions into one measure, depending on the philosophical perspective taken, and there is little agreement amongst social scientists on which method to use (Hagerty and Land 2007). Each approach will result in different scalar measures of quality of life, and will lead to different policy implications, for example country rankings (Nardo et al. 2005) and identification of people classified as “worse-off” in their society.

The OECD has issued clear guidelines on how to construct composite indices, and they encourage documenting and explaining the weighting and aggregation procedures selected (Nardo et al. 2005). However, despite Sen and Anand’s argument that the choice of weights be open to questioning and debate in public discussion (Anand and Sen 1997); several available measures of wellbeing do not make the value judgments and the aggregation method explicit and thus cannot be open to public scrutiny on what a good life should look like. Hagerty et al. (2001) review over 20 quality of life indices and conclude than none adequately addresses the issue of weighting.

This paper aims to contribute to the literature on the implications of adopting different methods for setting the weights in composite measures of wellbeing. Two questions are addressed:

1. Does it matter how the dimensions of a wellbeing index are aggregated?
2. What are the implications for the identification of the “worse off” in a society?

Four weighting techniques are used for the aggregation of a multidimensional measure of wellbeing based on Sen’s capability framework: the Women’s Capabilities Index. The index has been developed for assessing women’s quality of life in Mchinji District, Malawi (Greco et al. 2015), as part of Maimwana Project, a community based programme aimed to reduce maternal and neonatal mortality in the area (Lewycka et al. 2010).

## Setting the Weights in Wellbeing Indices

The Oxford Poverty and Human Development Initiative (OPHI) distinguishes three classes of categories for setting the weights in multidimensional measures of wellbeing that are based on different theoretical assumptions (Decancq and Lugo 2012):

- Normative: equal/arbitrary and expert-based approaches.
- Data-driven: Statistical techniques (e.g. principal component analysis, factor analysis, latent variable models).
- Hybrid: Survey-based methods to elicit directly people’s preferences (standard gamble, visual analogues, and willingness to pay) or making use of subjective wellbeing surveys.

### Normative

Normative approaches are based on value judgments of a specific group of people, which can include the researcher, a panel of experts, the wider community or the participants in the study. The easiest and most common approach for setting the weights in multidimensional measures of wellbeing is to assume equal value for each dimension. Examples of this include the Human Development Index (Anand and Sen 1997), the Human Poverty Index and the Gender-related Development Index (UNDP 2013), and the OPHI Multidimensional Poverty Index (Alkire and Foster 2011). Lorgelly et al. (2008) assigned an equal weight to each of the 18 questions of their survey. For example, being able to live a life of normal length is as equally important as being able to enjoy recreational activities and being capable of independent thinking.

This approach has been defended for its simplicity, for its ‘agnostic’ viewpoint (Decancq and Lugo 2012), and for minimising disagreement even amongsts very different individuals (Hagerty and Land 2007). However, there are many criticisms for its lack of explicit value judgments (Ravallion 1997), and it seems unrealistic to assume that all capabilities are equally valuable to people: “obviously convenient but also universally considered wrong” (Chowdhury and Squire 2006, p. 762).

Besides the equal weight approach, there is a range of more complex aggregation techniques based on people’s values or expert opinions. Sen advocates for “open discussion, debate, criticism and dissent” as the means for eliciting values and priorities: “We cannot, in general, take preferences as given independently of public discussion.” (Sen 1999 p. 7). People’s values can be elicited with participatory methods such as the budget allocation technique: people are asked to distribute a budget of points to each dimension, the higher the importance of the dimension, the higher the number of points (Chowdhury and Squire 2006). The OECD Better Life Index by default sets equal weights to the eleven dimensions; however, the index is an interactive tool available on the OECD website, and users are allowed to set their own weights on a scale from 1 (least important) to 5 (most important). The ranking of the 33 OECD members’ countries changes accordingly to the user’s value judgment and this is then presented in a powerful graphic (OECD 2011).

### Data-Driven

Statistical approaches depend only on the distribution of specific achievement levels in society; they are not based on any value judgment and given the fact that the researcher has no influence on the weights that are used, they are regarded as a more objective way of determining weights (Boelhouwer 2010). However, some have argued that there is always a normative element into objective indicators, and measures free of value judgments are, in practice, impossible to create (Cobb and Rixford 1998; Diener and Suh 1997; Booysen 2002).

These techniques are based either on descriptive or explanatory models.

The most common descriptive models are principal component analysis (PCA) and factor analysis (FA). They are a set of multivariate statistical techniques that help to extract information from the data; they are conceptually very similar, and they share the same aim: to facilitate multidimensional analysis by reducing the number of variables and therefore reducing the complexity. However, there are some differences in the way this aim is achieved. PCA does it “describing the data” and FA “estimating latent variables”. FA is a more flexible model than PCA (it has more parameters) and can often be more useful. As explained in Bartholomew et al. (2002), PCA is a descriptive technique which does not assume an underlying statistical model, which Factor Analysis does. Depsite the differences, PCA can be a good approximation to factor analysis, and in fact it can even be regarded as a method of factor analysis (Bartholomew et al. 2002).

PCA is commonly used in the development of socio-economic status and wealth indices (Vyas and Kumaranayake 2006; Howe et al. 2008). Factor analysis has been also widely employed (Noble et al. 2000; Schokkaert and Van Ootegem 1990) but involves some challenges. First, if the observable variables submitted to factor analysis are measured on different scales, the factors might pick up method effects rather than substantive variance effects. Secondly, it is uncertain that if the real life functionings are correlated, orthogonal factors would represent adequately an individual’s welfare (Decancq and Lugo 2012).

Other indices have been constructed using the nonlinear canonical correlation analysis method, which is a variation of principal component analysis. It calculates the weights in a way that the item total correlation is maximised. The advantage of this method is that it can work with different types of measurement. The indicators do not have to be measured at ordinal or interval level but nominal indicators can also be included (Boelhouwer 2010).

More complex and sophisticated aggregation approaches are explanatory models such as latent variable models (which is a factor analysis for categorical data), structural equation models and fuzzy set theory (Chiappero Martinetti 1994; Di Tommaso 2006; Krishnakumar 2007) These are based on the assumption that the indicators are dependent on a set of unobservable latent variables (e.g. quality of life). These probabilistic models are not straightforward to interpret (Bartholomew et al. 2002) and as a result can be said to lack transparency in terms of facilitating a clear understanding for policy makers and interested individuals who may be interested to use the findings.

Regression analyses have also been used to derive weights. However, they are very different from the above methods because they look at how well one variable (e.g. life satisfaction) can be estimated by other variables and the aim is not to reduce the number of items.

### Hybrid

The hybrid approach combines people’s opinions with quantitative analysis. Survey-based approaches to weighting have employed standard methods from economics for eliciting preferences, such as discrete choice modelling (DCE) (Coast et al. 2008; Watson et al. 2008). Coast and colleagues valued the ICECAP index for older people using best-worst scaling along with a stated preference discrete choice experiment (BWS DCE). Because the respondent is asked what attribute is the best or the worst, and the respondent does not have to trade one for the other, Coast argues that it can be considered as a value judgment rather than a choice (Coast et al. 2008). Moreover, this type of DCE is better than traditional DCE because it gives more information on preference heterogeneity rather than ‘pick one’ choices, and is less cognitively demanding (Flynn et al. 2007). However, it has been argued that BSW DCE is more similar to a standard elicitation method than the capability framework (Cookson 2005). Other recommended methods include the use of vignettes (Lorgelly et al. 2008), the multi-attribute utility method (Kinghorn et al. 2015) and survey ranking (De Kruijk and Rutten 2007).

## Overview of the Women’s Capabilities Index

The Women’s Capabilities Index was developed with a number of steps: (1) development of a theoretical framework (selection of dimensions relevant to the study and context); (2) development of a measurement model (selection of indicators and questionnaire design); (3) building the capability set (survey); (4) weighting and aggregation; and (5) validation of the index.

The theoretical framework was developed with a participatory technique (focus groups) as described in Greco et al. (2015). It was based on women’s contributions to the conceptualisation of a good life which were analysed and grouped by the researcher into a set of six main dimensions, or capabilities, of which each had a set of subdimensions. Based on the conceptual framework, a measurement model was developed.

A protocol describing in lay terms the theoretical foundation of the index was presented and discussed with the local research team, with a proposition of a measurement model based on the re-elaboration of the lists of capabilities that were identified during step 1. With the adoption of the capability approach, each “being and doing” that was valued by women as important in their lives, regardless of its achievement, was considered part of the measure and no external value judgment was introduced in the development of the index.

For example, the focus group participants mentioned being happy (cheerful) and contented with life as important “ingredients” of a good life. Happiness was not considered the aim or purpose, or final goal of life. Hence, happiness is considered one dimension amongst others. It was measured using two indicators (that make up to one dimension): happiness, and life satisfaction, to reflect the complexity of its meaning.

The measurement model comprised six sections related to the capabilities, or wellbeing dimensions, derived from people’s values: *physical strength, inner wellbeing, household wellbeing, community relations, economic security, and happiness*. Each dimension comprised a set of sub-dimensions for a total of 26 sub-dimensions. The sub-dimensions of the model were assessed with different indicators for a total of 72 variables (Table 1).

**Table 1:** Structure of the wellbeing measure

*Physical strength* 4 sub-dimensions, 9 variables
Being able to do physical work: physical health, energy
Having enough food: types of food eaten in the last week
Being able to avoid diseases: hygiene, HIV awareness, HIV protection, bed net use
Being able to space births: family planning availability, FP practice
*Inner wellbeing* 5 sub-dimensions, 11 variables
Peace of mind: mental health, sleep lost, relax time
Control over personal matters: control over daily activities, permission to go to funeral, permission to go to clinic
Free from oppression: freedom of expression, lack of oppression
Living without shame
Knowledge: read, write
*Household wellbeing* 5 sub-dimensions, 13 variables
Free from domestic violence: domestic violence past, domestic violence likely in future
Control over money: access household money, control over minor expenditure, control over major expenditure
Living in a decent house: toilet, water, house tenure, fear of house eviction, house adequate, house adequate in 6 months
Children’s education: all children will reach desired level of education
Family care: take care of children and husband
*Community relations* 5 sub-dimensions, 21 variables
Access services: easy/difficult to reach health centre, under 5 clinic, school, market, water source, church
Feeling safe and comfortable in the village: fear of witchcraft, moving away from village, safety village, assault past, assault future, theft past, theft future
Being able to join community groups: groups available, group membership, position
Social exclusion and discrimination: not allowed in groups, gender discrimination, poverty discrimination
Being respected: respect, admiration
*Economic security* 5 sub-dimensions, 16 variables
Safety net: help asked to you, you asked for help
Land: land ownership, fear of eviction,
Assets: bike, oxcart, ox, chicken, pig, goat, cow, radio, mobile, bed net
Business opportunities: access to business opportunities
Copying with shocks: able to feed the family if crisis
*Happiness* 2 sub-dimensions, 2 variables
Satisfaction: satisfied with life overall
Happiness: taking all things together, how happy are you?

Based on the measurement model, a survey tool was developed in order to collect data on the capabilities of a sample of women in Mchinji District, Malawi. The instrument was extensively piloted and tested for content validity, construct validity and reliability (Greco 2013).

## Methods

### Household Survey

The data were purposively collected from a household survey conducted between March and June 2010 in Mchinji District, Malawi, on a sample of women.

The objective of the sampling was twofold: to gain representativeness of the study area and to compare, in a subsequent study, the quality of life of women who had been exposed to the MaiMwana Women’s group intervention with women living in control areas. The sample included 6 out of 48 clusters of the MaiMwana Project trial (Lewycka, Mwansambo et al. 2010): 3 clusters with the intervention Women’s Group only and 3 control clusters (no interventions). The clusters were randomly selected. A total of 345 women who had delivered in the previous year were randomly selected from the surveillance register of participants enrolled in the main trial:

- 115 women from control clusters,
- 115 women from women’s group clusters who participated in at least one Maimwana women’s group meeting,
- 115 women from women’s group clusters who had never participated in a Maimwana women’s group meetings.

The survey was administered by two local fieldworkers trained on social research methods. Households were randomly allocated between interviewers and the share was even (52 and 48%). The non-response rate in the survey was 25%: 78 (23%) women were not located for different reasons: moved village, died or were misclassified in the surveillance register; 9 (3%) women were located but were not available for the interview despite a second visit. The mean duration of the interview was 48 min (95% CI: 47–50). All data cleaning and analyses were performed with Stata version 12.

### Aggregation

Drawing on the work of OPHI (Decancq and Lugo 2012), four methods have been used for setting the weights for the Women’s Capabilities Index, one from each class of category, plus the equal-weight approach. The four methods are:

1. *Equal* the dimensions have equal value
2. *Normative* the weights are drawn from a participatory exercise based on collective value judgments
3. *Hybrid* the weights are derived from survey-based individual preferences
4. *Data* the weights are set using principal component analysis (PCA)

In the literature, the equal weight approach is classified as a normative aggregation method. However, to avoid confusion in this paper, the normative approach will only signify the participatory technique and, given its ‘agnostic’ view point, the equal weight is considered a separate category.

The dimensions in the equal-weight index (1) have been assigned equal value. The weights are calculated as the arithmetic mean across sub-dimensions and across dimensions.

The weights for the normative index (2) are based only on normative value judgments. They were derived from a deliberative democratic process (Burchardt 2012), consistent with Anand and Sen’s argument for public debate and scrutiny in setting the weights (Anand and Sen 1997; Sen 2004; Alkire 2005). A series of focus groups were held with women of reproductive age in Mchinji District, Malawi with the twofold aim of selecting the capabilities for a “good life” and eliciting the values of each capability in a participatory manner. After having identified the relevant dimensions of quality of life, focus group participants discussed the relative importance of the dimensions, made partial-ordering and finally reached an agreement on the values, assigning up to ten beans for each dimension (ten beans was the maximum value, no trade-offs allowed). The weights were derived from the arithmetic mean of the bean-value assigned to each dimension across the different focus groups. The mean values were then normalised to a 0–1 scale.

The weights for the hybrid index (3) were derived from a combination of value judgments and statistical distribution. Individual preferences were elicited through the household survey. In the survey, respondents were asked to rank the dimensions from 1 (most important) to 6 (least important) according to their opinion. The method adopted for moving from ranking to weights has been used in the multidimensional poverty literature (De Kruijk and Rutten 2007). First, a group ranking is calculated as the mean of individual rankings. The weight for dimension *j* is then determined by the following formula:

$$w_{j} = \frac{{1 + d - r_{j} }}{{1 + d - \mathop \sum \nolimits_{j = 1}^{d} r_{j} }}$$

where *d* is the number of dimensions and *r*_*j*_ is the ranking of dimension *j* with value 1 if it is the most important, 2 if it is the second most important dimension and so on.

For the normative (2) and hybrid (3) indices, the sub-dimensions were aggregated within each dimension using the arithmetic mean, as done in the OECD Better Life Index (OECD 2013). The six dimensions were then aggregated using the weights generated with the two different methods.

For generating the data-driven index (4), principal component analysis (PCA) was applied to the raw dataset and the factor loadings of the first component were used to predict the score for each individual in the sample. Amongst the data-driven methods, PCA has been chosen because it is an efficient and well understood descriptive statistical technique for determining weights for components of poverty and wealth indices (Noorbakhsh 1998; Klasen 2000; Filmer and Pritchett 2001).

### Comparison

The indices were compared graphically, with correlation coefficients, across quintiles, and against a pre-defined benchmark. Given its agnostic nature, the equal weight approach was used as comparator. In addition, the indices were compared to a standard wealth measure.

In order to make a meaningful comparison, the values of the four indices were normalised. As Coast highlights (Coast et al. 2008), integrating dimensions raises concerns about the meaning of anchoring at death as is done in the QALY. Being alive is a pre-condition for enjoying any type of capability; however the debate remains over whether the absence of capability is equal to death, or worse than death.

The normalisation was done according to a standard function which converts the original values of the indicators into numbers varying in a range between 0 (for the worst possible outcome) and 1 (for the best possible outcome), without affecting the distribution. The transformation formula used was:

$$I_{i}^{\prime } = {{\left( {I_{i} - \hbox{min} } \right)} \mathord{\left/ {\vphantom {{\left( {I_{i} - \hbox{min} } \right)} {\left( {\hbox{min} - \hbox{max} } \right)}}} \right. \kern-0pt} {\left( {\hbox{min} - \hbox{max} } \right)}}$$

where* I*′_*i*_ is the rescaled score of the individual *i*, *I*_*i*_ is the original score of the individual *i*, and min and max are respectively the minimum and maximum values of the original indicator scored in the sample.

The distribution of each index was examined and compared graphically to assess the extent of skewness. The degree of correlation between the indices was estimated using the Pearson product-moment correlation coefficient. In addition, the correlation of the rankings of the population was explored with the Kendall tau rank correlation coefficient. The data is ranked in ascending order with the equal index (1) as reference. Two Kendall tau correlation coefficients were estimated to measure the association (similarities of ordering) between the different indicators. The Kendall τ coefficient is defined as:

$$\tau = \frac{{\left( {{\text{number}}\,{\text{of}}\,{\text{concordant}}\,{\text{pairs}}} \right) - \left( {{\text{number}}\,{\text{of}}\,{\text{discordant}}\,{\text{pairs}}} \right)}}{{\tfrac{1}{2}\, n\left( {n - 1} \right)}}$$

The coefficient is expected to be in the range −1 ≤ τ ≤ 1. If the agreement between the two rankings is perfect (i.e., the two rankings are the same) the coefficient has value 1. If the disagreement between the two rankings is perfect (i.e., one ranking is the reverse of the other) the coefficient has value −1. If the indicators are independent, the coefficient is expected to be approximately zero.

### Analysis Across Quintiles and Against a Deprivation Threshold

In order to facilitate comparison, the population was divided into quintiles according to their index scores and indices were compared to each other in terms of misclassification of individuals in quintiles. Individuals were grouped into 5 quintiles of 48 or 49 people each according to the ascending value of the indexes. Kappa statistics were calculated in order to assess the agreement of classification between indices. The Kappa statistic is a measure of reliability that takes into account the agreement expected on the basis of chance. A Kappa statistic of 1 indicates perfect agreement and a value of zero indicates no agreement better than chance. In general a Kappa value of <0.5 indicates poor agreement (Howe et al. 2008).

In addition to the misclassification of quintiles, the distribution of the indices’ scores was also investigated using a predefined threshold for the identification of individuals as “worse off” or “better off”. A measure of relative deprivation as opposed to absolute deprivation is more appropriate for comparing the wellbeing scores in a given population. An arbitrary cut off point was set at 60% of the median value of the index. This threshold is the internationally agreed measure of relative deprivation used throughout the European Union (Atkinson et al. 2004) and it was chosen for its simplicity, transparency and straightforward interpretation: the individuals who fell below the threshold were the ones who scored <60% of the median score in the capability index.

### Comparison with a Wealth Index

The literature is rich in attempts made to compare different measures of wealth and deprivation; and the difference between the classification of income and other dimensions of wellbeing has long been noted (Atkinson 1983; Klasen 2000; Ruggeri Laderchi et al. 2003; Kingdon and Knight 2006). The work of OPHI brings extensive empirical evidence to bear on the mismatch of income-related indicators and multidimensional measures for the identification of people living below a deprivation threshold in society (Alkire and Seth 2013). Ruggeri Laderchi et al. (2003) examine and compare different approaches to poverty. They show empirically that there is a considerable lack of overlap between individuals falling into income deprivation and capabilities’ deprivation.

To further contribute to this growing literature, each index in this study was compared with a conventional measure of wealth. The aim was to assess the extent of divergence between a measure of deprivation based on capabilities with a more conventional approach based on socio-economic status.

Asset indices are often used for estimating people’s socio-economic status, thanks to several comparative advantages they have over income or expenditure measures. Collecting accurate income data is time-consuming and difficult especially if large sectors of the economy are informal, goods are traded with goods, seasonality is high and income is produced from different sources (Montgomery et al. 2000; McKenzie 2005; Vyas and Kumaranayake 2006). Expenditure data are more reliable and easier to collect compared to income data (Howe et al. 2008), but they still require extensive (and expensive) fieldwork. For these reasons, using information on household assets derived from the survey, an asset index was built using principal component analysis. A two-fold approach was used to select and retain the asset variables (Borghi 2006).

1. All asset variables for which data were available were included, regardless of the variation between households (Gwatkin et al. 2007).
2. Assets were retained on the basis of their factor loading (Booysen et al. 2008).

Based on the survey, the following variables were available: type of water source, type of toilet, land ownership, house ownership, type of roofing material, bike, oxcart, ox, chicken, pig, goat, cow, radio, mobile and bed net. The assets with a factor loading smaller than 0.20 were dropped (Borghi 2006). The assets retained and included were: type of toilet, type of roofing material, bike, oxcart, ox, chicken, pig, goat, cow, radio, mobile, bed net. Principal component analysis (PCA) was applied to these twelve variables and subsequent scores derived.

## Results

### Socio-Demographic Characteristics

The socio-demographic characteristics of the respondents are presented in Table 2, with national statistics of women aged 15–49 for comparison. Almost all respondents (over 94%) lived in rural areas (national: 81%). Almost 78% of women were younger than 35. The mean age was 29 years, with one woman younger than 16 years and 5 women aged over 50 years. The majority of women (around 85%) were married or lived with a partner (national: 67%). The dominant religion was Roman Catholic (63%, national: 20%) and the dominant tribe was Chewa (89%, national: 34%). Over 83% of respondents declared that their husband or partner was the head of the household. More than 65% of families had 3 or more members below the age of 15, and only 13% had 1 or 2 members above the age of 50. More than half of the respondents were able to read (national: 68%), although only 11% had completed secondary school (national: 18%). The vast majority of the respondents (over 88%, national: 58%) were agricultural farmers. The socio-demographic characteristics are in line with national statistics for women of reproductive age with the exception of religion and ethnic group.

**Table 2:** Socio-demographic characteristics of the respondents (n = 258)

Variable	Values	Frequency	Sample (%)	Malawi (%)^a^
Area	Rural	242	94.2	81.3
	Peri-urban	15	5.8	18.7
Age (approx)	<16	1	0.4	–
	16–20	20	7.8	21.7
	21–25	93	36.1	19.8
	26–35	87	33.7	33.2
	36–45	52	20.2	18.5
	46–55	5	1.9	6.8
Status	Married or with partner	219	84.9	67.4
	Single or never married	3	1.2	19.7
	Divorced	33	12.8	9.3
	Widowed	3	1.2	3.6
Religion	CCAP^b^	23	9.0	16.6
	Roman catholic	161	62.7	20.6
	Anglican	6	2.4	2.3
	Pentecostal or adventist	48	18.7	6.7
	Other	19	7.4	53.8
Tribe	Chewa	230	89.2	34.1
	Ngoni	24	9.3	12.9
	Other	3	1.6	53
Household headship	Yourself	30	11.6	
	Husband or partner	215	83.3	
	Mother or father	12	4.7	
	Other	1	0.4	
HH members under 15	1 or 2	89	34.5	
	3 or 4	135	52.3	
	5+	34	13.2	
HH members over 50	0	225	87.2	
	1	26	10.1	
	2	7	2.7	
Education	Never been to school	36	14.0	15.2
	Primary	193	74.8	64.8
	Secondary	29	11.2	18.1
	More than secondary	0	0.0	1.8
Read	Yes	151	58.5	67.6
	No	107	41.5	33.4
Employment	Farmer	228	88.4	57.8
	Trader	17	7.0	24.7
	Other/occasional job	13	6.0	38.5

^a^Malawi DHS 2012: Percent for women aged 15–49, national

^b^Church of Central Africa Presbyterians

**Fig. 1:**
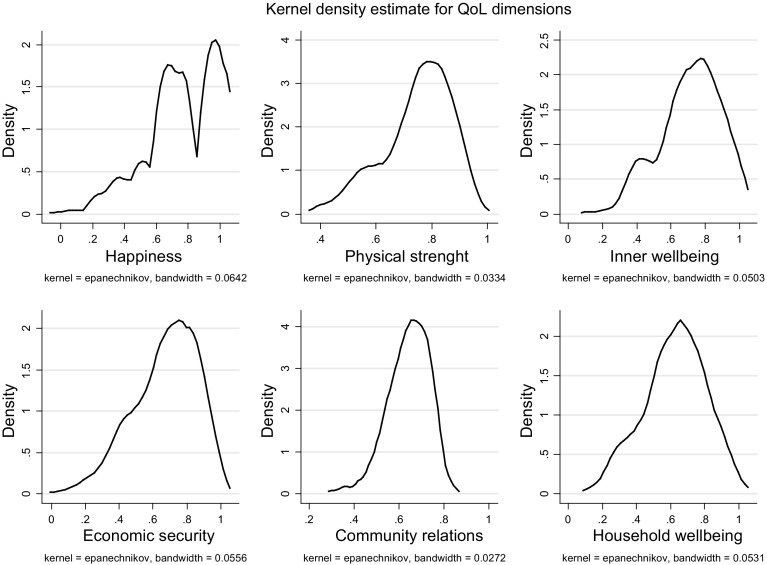
Kernel density curves of the quality of life dimensions

The distribution statistics of the non-aggregated dimension scores are presented in Table 3, in descending order sorted by mean value. The *happiness* dimension had the highest average score, followed by *physical strength, inner wellbeing*, and *economic security*. *Community relations* and *household wellbeing* had the lowest scores. The Fig. 1 shows the density curve estimates of the dimensions. With the exception of *happiness,* all dimensions appear to be normally distributed. Happiness has a different shape probably because it is made up of only two indicators, compared to the other more complex dimensions.

**Table 3:** Dimension scores distribution statistics

Dimensions of quality of life	Min	Max	Mean	SD
Happiness	0.00	1.00	0.77	0.22
Physical strength	0.39	0.97	0.75	0.12
Inner wellbeing	0.13	1.00	0.71	0.18
Economic security	0.05	1.00	0.67	0.19
Community relations	0.31	0.84	0.64	0.09
Household wellbeing	0.14	1.00	0.62	0.18

### Aggregation

Four indices were created using the following aggregation methods:

1. *Equal* the dimensions have equal value.
2. *Normative*: the weights are based on collective value judgments from the focus groups.
3. *Hybrid* the weights are derived from survey-based individual preferences.
4. *Data* the weights are set using PCA.

The weights derived from the equal, normative and hybrid methods are presented in Table 4 sorted by the normative weights in descending order. For the equal and hybrid approaches, the sum of the weights is 1. For the normative, the weights function as a deflator of the value of the dimensions, and hence do not sum to 1. The final index is calculated as the weighted average of the dimensions. The weights for the data driven method are presented in a separate table (Table 5) because they are assigned to each variable rather than to the dimensions.

**Table 4:** Weights for equal, normative and hybrid approaches

Dimension	Weight		
	Equal	Normative	Hybrid
Physical strength	0.17	0.95	0.29
Household wellbeing	0.17	0.94	0.19
Community relations	0.17	0.94	0.14
Happiness	0.17	0.88	0.05
Inner wellbeing	0.17	0.87	0.24
Economic security	0.17	0.84	0.10
Total	1.00		1.00

**Table 5:** Weights for data-driven approach (five higher)

Indicator	Weight
Being able to cope with shocks	0.25
Being able to take care of the family	0.20
Being happy	0.19
Being admired	0.19
Having a bed net	0.18

The weights for the data-driven index were derived from the first component of the PCA. 100 components were generated; the first component (that explains the largest variance) explained only 8.3% of the variance. The highest five weights are presented in Table 5. The indicator *being able to cope with shocks* was assigned the highest weight, followed by *family care, being happy, being admired* and *having a bed net.* The scale reliability coefficient is 0.84, suggesting that the variables were highly correlated.

The dimension for *physical strength* was given the highest weight in the normative and hybrid indices. *Economic security* was assigned a relative low value in both normative and hybrid indices, however the PCA assigned the highest weight to one of the components of this dimension. *Happiness* scored the lowest weight in the hybrid approach setting, however it was one of the top five variables in the PCA.

Table 6 presents the mean, standard deviation, minimum and maximum values of the indices. While the standard deviation was very similar for the four indexes, the mean value of the data driven index differed greatly from the mean values of the other three indices.

**Table 6:** Comparison of means, standard deviation, minimum and maximum values of the rescaled indexes

Index	Mean	SD	Min	Max
Equal	0.67	0.18	0	1
Normative	0.66	0.19	0	1
Hybrid	0.64	0.18	0	1
Data-driven	0.29	0.20	0	1

### Distribution and Correlation of the Indices

**Fig. 2:**
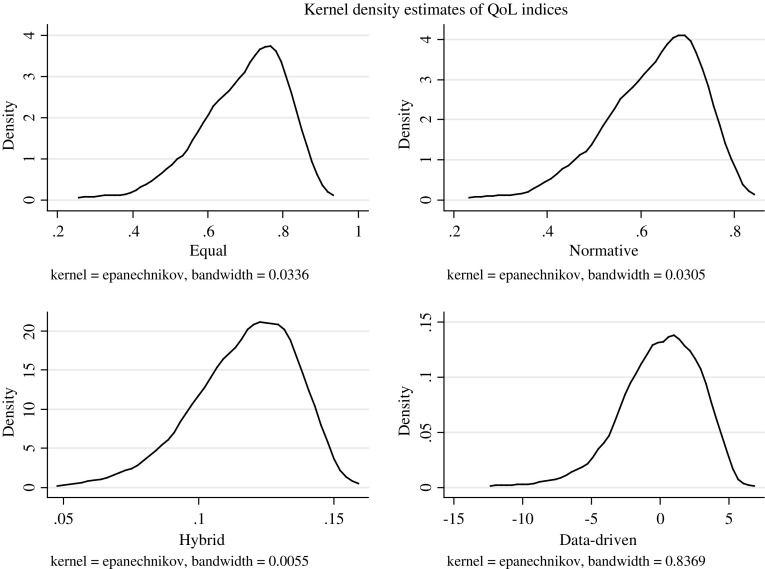
Kernel density curves of the indices

The Kernel density curves of the four indices are plotted in Fig. 2. The graph suggests that the population was similarly distributed across the four indices, and slightly skewed at the right end with a long left tail, suggesting that there was a smaller number of people who were worse off versus a larger group which enjoyed better quality of life.

The Pearson product-moment correlation coefficients suggest that the indices are highly correlated (Table 7); the data-driven index shows a less strong correlation coefficient compared to the others but still very close to 1 (perfect correlation).

**Table 7:** Pearson product-moment correlation coefficients for the four indices

	Equal	Normative	Hybrid	Data-driven
Equal	1			
Normative	1	1		
Hybrid	0.97	0.97	1	
Data-driven	0.90	0.90	0.87	1

Similar results are suggested by the rank correlation coefficient. As Table 8 shows, the ranking of the data driven index is the one that differs the most to the ranking of the equal weight approach, compared to the other indexes.

**Table 8:** Kendall tau rank correlation coefficients for the four indices

	Equal	Normative	Hybrid	Data-driven
Equal	1			
Normative	0.99	1		
Hybrid	0.83	0.84	1	
Data-driven	0.70	0.70	0.66	1.00

### Analysis Across Quintiles

Table 9 shows the movement across quintiles of individuals in the equal weight index compared to the other indices. The normative approach index has perfect agreement of classification in quintiles with the equal weight index: individuals remained in the same quintile regardless of the use of either measure. The value of Kappa statistics confirmed this.

**Table 9:** Movement of individuals between quintiles of the equal index and quintiles of the other indices

% Individuals moving between quintiles	Normative	Hybrid	Data
Same quintile	100	72.84	55.14
Move one quintile	–	26.34	37.83
Move two quintiles	–	0.82	7.01
Move three quintiles	–	–	–
Move four quintiles	–	–	–
Kappa	1*	0.66*	0.44*

* *p* < 0.001

Comparing equal weight and hybrid approach, 73% of respondents remained in the same quintile, with a kappa statistic of 0.66. Less than 1% of people were estimated to drop (or to increase) by two quintiles.

The flow of people across the groups was more significant when compared to the data driven approach, where nearly 45% of people were misclassified. For example, a small number of women were assigned to the middle quintile with the equal-weight index; however, if the data driven approach was adopted, they were in the bottom 20%. The value of Kappa statistics was 0.44.

### Analysis Against a Deprivation Threshold

The people who fell below the deprivation threshold were estimated to be 10–11% of the population, if the equal, normative and hybrid approaches were adopted. If the data-driven approach was used, than the “worse off” were <7%. If the wealth index was used, than the individuals who fell below the threshold were estimated to be around 28% of the population.

The equal weight and normative approaches yielded very similar estimates of the deprived population, with less than a 1% difference. The hybrid method also had similar estimate, with a 13% more people classified as “worse off” compared to the equal value index.

The data-driven index failed to capture 44% of deprived people that were so classified by the equal value index.

The “worse off” classification of the asset index was consistently different from the equal value index: an additional 21% of the total population fell below the deprivation line if the asset index was used, compared with the equal value measure.

The same 10% of the total population were estimated to fall below the deprivation line in the three approaches (equal, normative and hybrid). Together with the asset and data-driven indexes, the same 6% of the population fell below the threshold regardless of the measure adopted. All of them were in the bottom quintile however the groups were formed.

Taking a closer look at the people who lived below the threshold regardless of the measure used, it is worth noting that half of them were in good health or with minor health issues that were not affecting their daily activities (sample: 70%). The majority of them (86%; sample: 42%) were not able to read or write; almost 60% (sample: 35%) had been a victim of domestic violence, with 29% (sample: 10%) reporting frequent assaults. 64% (sample: 33%) were feeling oppressed to some extent and almost 80% were feeling ashamed or inadequate (sample: 33%); 57% (40%) reported that they did not have total freedom over personal decisions nor had access to household money without permission from the husband or from somebody else. 64% (sample: 47%) asked for assistance in terms of money or food during the last year, and the remaining 36% (sample: 12%) were too ashamed to ask for it. None of them had been asked for assistance (sample: 55%). All of them had access to a piece of land, with 71% of them (sample: 94%) declaring being the land owner, although none of them was confident that she would be able to cope with a shock such as a failed crop (sample: 65%). Finally, 64% (sample: 7%) declared that, taking all things together, they were not satisfied with their lives, and only 21% (sample: 87%) reported being fairly happy.

### Comparison with a Wealth Index

An asset index was created for each individual in the sample using PCA. The first component of the PCA, which is assumed to represent the wealth status of the individual, explained 31% of the variance in the data. This percentage is high compared to the range from 12 to 27% presented in the review of socio-economic status indexes conducted by Vyas (Vyas and Kumaranayake 2006). The scale reliability coefficient was acceptable at 0.78, suggesting that this index is a robust measure of socio-economic status.

The graph below depicts the distribution of the wealth across the study population. The distribution is skewed towards the left, suggesting that there is a high number of individuals with a lower socio-economic status versus a small number of richer people. This distribution is as would be expected with an income distribution.

**Fig. 3:**
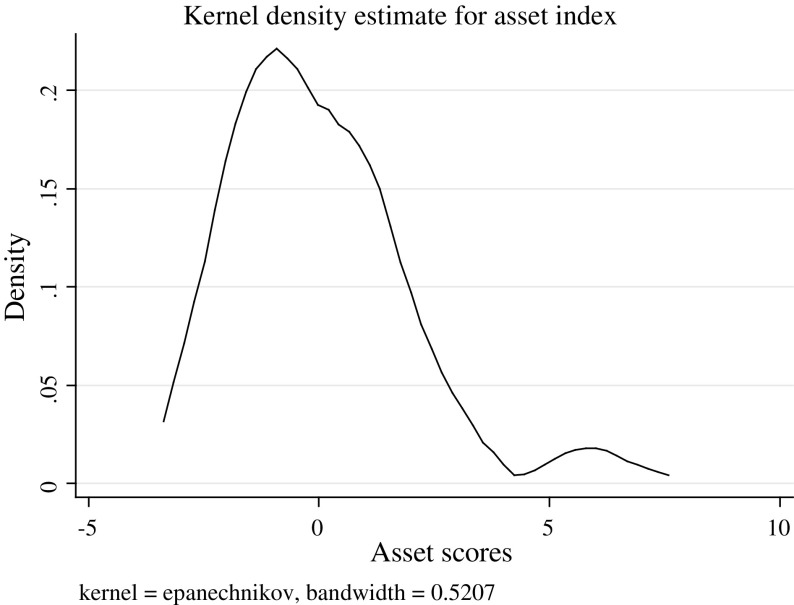
Distribution of the asset index

As Figs. 2 and 3 show, the distribution of the wealth index differed greatly from the distribution of the quality of life indices.

Findings from the analysis across quintiles showed that, relative to the wealth index, between 60 and 65% of people were misclassified in terms of their socio-economic position if one of the capability indices was used. A small group of people were assigned to the first quintile with one measure and to the bottom quintile with the other indicators (and vice versa). The value of Kappa statistics ranged from 0.19 to 0.25 (Table 10).

**Table 10:** Movement of individuals between quintiles of the asset index and quintiles of the other indices

% Individuals moving between quintiles	Equal	Normative	Hybrid	Data
Same quintile	35.12	34.98	38.68	40.33
Move one quintile	38.03	37.86	33.33	41.98
Move two quintiles	19.53	19.34	20.16	15.23
Move three quintiles	7.39	7.41	7	2.47
Move four quintiles	0.41	0.41	0.82	–
Kappa	0.19*	0.19*	0.23*	0.25*

* *p* < 0.001

## Discussion

The findings suggest that women did not give the same value to the different dimensions of quality of life. As the normative and hybrid approaches showed, respondents were able to make a value judgment, giving a higher value to those capabilities that were considered more important.

*Physical strength* had the highest value in both normative and hybrid indices, implying that having an able and strong body, being free from disease, having a choice in matters of reproductive health and having enough energy to work were regarded as the most important aspects in one’s life. *Happiness* scored relatively low compared to more fundamental measures of survival. Finally, despite the vast majority of the interviewed people being subsistence farmers and would probably be classified as extremely poor by the World Bank’s threshold of US$1.25 a day, *economic security* was the lowest priority in the participatory exercise, and the second lowest using the survey-based method. Economic resources, while important, were not all that mattered for women’s wellbeing.

Nonetheless, the equal value approach and the normative weights were highly correlated and had similar distributions. This might be because during the participatory focus groups, women assigned high values to all of the dimensions (from seven beans upwards), and in some discussions all the dimensions received a value of ten. This could suggest that all dimensions were considered highly and equally important for achieving “a good life”. There may well be a certain level of interdependence between the dimensions which made it difficult for someone to imagine the relative importance of say *community relations* in the absence of *physical strength*.

The data-driven index appeared to be the most different from the equal weight approach when tested with the kendall tau coefficient. This suggests that women would be ranked in a different way if the data-driven index is used for assessing their quality of life.

The difference between the capability indices and the socio-economic status measure was striking and builds on the findings of previous literature (Balestrino and Sciclone 2000). There was a substantial mismatch between income poor and the capability poor: the wealth index only included asset ownership and not other measures of wealth or wellbeing. The asset index only showed one aspect of the capability index, material wealth, and missed out the more complex aspects of what constitutes a good life. Moreover, people valued *economic security* as the least important dimension in the capability set.

The distribution of the asset index was different from the distribution of the other indices, probably because in the latter what was important to people was derived from a democratic deliberative process of what makes a good life, hence the majority of people met the criteria for a good life with few below that.

The aggregation method used for setting the weights in the multidimensional Women’s Capabilities Index did matter for establishing who were the “worst off” in society (especially if estimated with quintile distribution). Using a data-driven method, rather than a normative approach, made a substantial difference to identify the “worst off” in the population: only 65% of individuals remained in the bottom 20% regardless of the index used, and 56% of individuals remained in the top 20% regardless of the index used. This has great implications for policy aimed at improving the life of those at the bottom of society, as one of the first steps is to identify the people most in need.

The data driven method of letting the data decide how to put the weights does not allow for an independent and legitimate ranking of capabilities and there is no value judgement involved, which is a core component of Sen’s theory of freedom. The data-driven approach suggests two things: some dimensions are more diagnostic of the good life than others, and quality of life is multidimensional and complex; therefore cannot be accurately reflected through a simple sum score measure.

Evidence from both the normative and hybrid methods revealed that people were valuing dimensions as more or less important for them, therefore it is a misrepresentation to suggest that equal weights can be applied across all dimensions.

The validity tests showed that the survey-based valuation has an acceptable level of reliability but not with a high degree of correlation (Greco 2013). Moreover, the ranking exercise was found to be cognitively demanding, as the respondents had to rank in order of preference aspects of their lives that were considered all highly valuable and important. For this reason, this method would not be recommended.

Therefore, the normative method appears to be the more appropriate: in addition with being the approach closest to Sen’s philosophy for open discussion and public debate in the formation of values and priorities (Sen 1999), it was an effective deliberative democratic process in eliciting relative values for the different dimensions of wellbeing.

The high correlation and the matching quintile classification between the normative and the equal weight methods may give support to the idea that in a context of scarce resources and time constraints, the equal weight approach is no doubt the simplest solution: the estimates are likely not to differ substantially from people’s values hence it can be considered as a good proxy. This conclusion have also been suggested by other studies (Boelhouwer 2010).

This study faced a number of challenges. First, in the survey ranking, women commented that ordering dimensions from the most important to the least important was a difficult exercise. The results of the test–retest reliability described elsewhere (Greco 2013) reflect this challenge. Instead, the participatory valuation process in the FGDs proved to be very effective. However, it was important to ensure that the process was truly democratic and that each woman had the opportunity to express her opinion. For this reason, the moderator of the FGDs received further training on facilitation techniques, and the researcher observed each FGDs to ensure that the process was as inclusive as possible.

The second limitation is related to the rescale of the scores of the indices from a 0 to 1 value. An assumption was made to anchor the index to the absence of capabilities rather than to death. Although the normalisation did not affect the distribution of the indices, it is important to note that the absence of capabilities could be considered worse than death.

The third limitation concerns the choice of method for the data-driven index. The PCA has been chosen because it is a widely used and recognised technique for item reduction. However, the first component had low explanatory power (only 8.3%) implying that it would be very limiting to use only the first component of the PCA for explaining the variation of the data. The low explanatory power might indicate that the sample is homogenous; that there is little variance because people are similarly deprived, but it might also be due to the high number of variables included in the analysis and the complexity of correlations between variables (Vyas 2006). Thus, more sophisticated techniques such as structural equation models could offer a stronger platform for building the index.

The fourth limitation is related to the choice of deprivation threshold. The threshold was chosen for its simplicity and transparency in the interpretation of the results. The choice was arbitrary, and the results are likely to vary if a different deprivation line is set. However, the aim of the comparison of the indices against a predetermined benchmark was to assess the degree of misclassification of the people rather than the actual classification of the people as deprived or not. Hence, for the scope of this exercise, the choice is felt to be appropriate.

Finally, data on capabilities could suffer from social desirability bias; hence the distribution is skewed more towards the right compared to the objective socio-economic status measure. The interviewers were part of the MaiMwana Project research team and had much experience in administering surveys on sensitive issues in the study area. A lot of care was taken to ensure that the respondents felt at ease and comfortable when answering the questionnaires. This should have minimised the social desirability bias.

In this paper, the Women’s Capabilities Index is constructed using four different methods for setting the weights. The four approaches (equal value, normative, hybrid and data driven) were compared against each other and against a conventional wealth index. The results showed that the choice of aggregation had an impact on the classification of the individuals in quintiles and as “worse off” in the society. Thus, in the development of multidimensional wellbeing indices it is important to make the choice of weighting as explicit and transparent as possible.
